# Approximate Bayesian Computation for Estimating Parameters of Data-Consistent Forbush Decrease Model

**DOI:** 10.3390/e20080622

**Published:** 2018-08-20

**Authors:** Anna Wawrzynczak, Piotr Kopka

**Affiliations:** 1 Institute of Computer Sciences, Siedlce University, 08-110 Siedlce, Poland; 2 National Centre for Nuclear Research, 05-400 Swierk-Otwock, Poland

**Keywords:** modeling of the GCR transport, estimation of the diffusion coefficient parameter, bayesian computations

## Abstract

Realistic modeling of complex physical phenomena is always quite a challenging task. The main problem usually concerns the uncertainties surrounding model input parameters, especially when not all information about a modeled phenomenon is known. In such cases, Approximate Bayesian Computation (ABC) methodology may be helpful. The ABC is based on a comparison of the model output data with the experimental data, to estimate the best set of input parameters of the particular model. In this paper, we present a framework applying the ABC methodology to estimate the parameters of the model of Forbush decrease (Fd) of the galactic cosmic ray intensity. The Fd is modeled by the numerical solution of the Fokker–Planck equation in five-dimensional space (three spatial variables, the time and particles energy). The most problematic in Fd modeling is the lack of detailed knowledge about the spatial and temporal profiles of the parameters responsible for the creation of the Fd. Among these parameters, the diffusion coefficient plays a central role. We employ the ABC Sequential Monte Carlo algorithm, scanning the space of the diffusion coefficient parameters within the region of the heliosphere where the Fd is created. Assessment of the correctness of the proposed parameters is done by comparing the model output data with the experimental data of the galactic cosmic ray intensity. The particular attention is put on the rigidity dependence of the rigidity spectrum exponent. The proposed framework is adopted to create the model of the Fd observed by the neutron monitors and ground muon telescope in November 2004.

## 1. Introduction

The galactic cosmic rays (GCR) are the charged particles traveling from the outer interplanetary space into the heliosphere. During their travel through the interplanetary medium, GCRs are undergoing the influence (modulation) of the solar wind and the combination of the regular and turbulent interplanetary magnetic field (IMF) being the sources of various kind of drifts and diffusion of the GCR particles. The GCR stream reaching the Earth is constantly monitored by the world network of neutron monitors and muon telescopes (e.g., [1]). The changes in the GCR flux have different long and short scale quasi-periodic variations. The most known are the 22-years, 11-years and 27-days variations connected with the recurrent changes in the Sun activity. We also observe the short time (a few days) sporadic changes connected directly with the Sun activity and solar wind. A sudden decrease of GCR intensity and then its gradual recovery during 8–10 days is called the Forbush decrease (Fd) [2]. The Fds occur as an outcome of the substantial disturbances in the interplanetary space occurring due to the powerful coronal mass ejecta and solar flares on the Sun. The mechanism underlying the creation of the Fds has been studied by many researchers. The first papers [3] proved that the geomagnetic disturbances do not cause Fds. Later, the IMF enhancements [4] were indicated as the primary factor. Results presented in [5] suggested that reduced particle diffusion mainly produces the Fd in the turbulent region behind the shock. These results are supported by recent papers e.g., [6,7,8,9]. Data analysis presented in [6] demonstrated that the sheath region between the shock and the magnetic cloud, particularly the enhanced turbulent magnetic field, results in the scattering of cosmic-ray particles, and causes the following Fds. Authors recommend this as the most effective mechanism to produce a transient depression in cosmic-ray variations. In [7] they also showed that the enhancement of the IMF associated with Fds occurs mainly in the shock-sheath region, and the turbulence level in the magnetic field is also enhanced in this region.

One of the essential characteristics of the Fd is the dependence of Fd amplitude on the rigidity of GCR particles. This power law ($R^{-\gamma }$) characteristic is called the rigidity spectrum of the Fd. The study of rigidity dependence of the GCR intensity in different phases of the Fd was presented in [8,9,10] and references therein. Moreover, they established the relationship between the exponent γ of the rigidity spectrum of GCR variations and exponent ν of the power spectral density (PSD) of the IMF turbulence ($PSD=P{\left(\frac{f}{f_{0}}\right)}^{-\nu }$, where *P* is power, *f* is frequency and $f_{0}$ is normalization frequency) in the form γ∝2−ν [8,9,10]. This relation can be deduced based on the dependence of the diffusion coefficient $K_{\left|\right|}$ of GCR particles on the rigidity *R*, as $K\propto R^{2-\nu }$, where ν is an exponent of the PSD of the IMF turbulence (e.g., [11,12]). The relation between the exponents γ and ν entails an expectation of the rigidity dependence of the exponent γ arising from the changes in the exponent ν versus frequency. However, the Fd complexity makes difficult to reveal the dependence of γ on rigidity in detail. Reliance of the exponent γ of the rigidity spectrum of the Fd on the rigidity of GCR particles was presented for Fd on 9–23 September 2005 in [9] and for Fd on 5–21 November 2004 in [10]. Based on the neutron monitors data divided into two groups according to their cut-off rigidities, it was shown that the exponent γ is larger when larger are the cut-off rigidities of stations used in its calculations. Correspondingly, the consistent frequency dependence of the exponent ν of the PSD of the IMF components during the Fd was observed.

However, the restrictions in the experimental study do not allow to estimate the formula for the diffusion coefficient and at the same time specify the rigidity dependence of γ during the Fd. In such situations, modeling can be applied to verify if the proposed model results agreeing with the experimental observations. This kind of study is presented in this paper.

To describe accurately, the considered physical processes scientist employ the mathematical and statistical models which become more and more complicated regarding regulatory relationships and computational complexity. Therefore, effective and efficient methods are required to infer unknown parameters in these models in order to reduce the simulation errors against the experimental data. There are two significant categories of inference methods: the optimization methods and Bayesian statistical methods. The optimization methods are designed to minimize an objective function by searching for parameters within a given parameter space. These methods infer the set of parameters resulting in the best fit to the experimental data. The second class is the Bayesian inference methods, which can estimate the probability distributions of parameters by using the Bayes’ rule to update the prior probability estimates. The important is that Bayesian methods are more robust in dealing with stochastic models and/or experimental data with noise (e.g., [13]). Moreover, they converge quite quickly in higher dimensions. In recent years Bayesian methods have been successfully used in a diverse range of fields and provide the promise to applications [14]. Among them, the approximate Bayesian computation (ABC) becomes in the last years a popular in a wide range of inference problems in biology, economics, engineering and physical sciences. The recent advances in ABC provide effective methods without any restriction on the requirement of the likelihood function.

We have employed the ABC methods to sample the parameters of the diffusion coefficient $K_{\left|\right|}$ included in the Fd model. The formula proposed for diffusion coefficient takes into account the rigidity dependence of the exponent γ. The sampling is guided by the comparison of the modeled amplitudes of the Fd on the subsequent days with the Fd registered by the neutron monitors and ground muon telescopes on 5–21 November 2004 [10]. This approach allows us to estimate the most probable profile of the diffusion coefficient during the Fd, especially confirm/or reject the postulate of the rigidity dependence of the exponent γ of the rigidity spectrum of Fd.

The paper is organized as follows: Section 2.1 introduces the stochastic methodology used to model the Fd of the GCR intensity. Section 2.2 presents the ABC methodology and presents in details the algorithm developed to sample the parameters of the diffusion coefficient during the Fd. Finally, the results of the computations are given in Section 3 and summarized in Section 4.

## 2. Applied Methodology

### 2.1. Stochastic Model of the Forbush Decrease of GCR Intensity

We model the transport of GCR in the heliosphere by the stochastic approach. In this approach, the individual particle motion is described as a Markov stochastic process, and the system evolves probabilistically. Consequently, the Parker equation [15] describing the GCR particles transport in the heliosphere can be brought into a form of the backward Fokker-Planck equation as [16,17]:

(1) $$\frac{\partial f}{\partial t}=K{\vec{\nabla }}^{2}\cdot f+\left(\vec{\nabla }K-\vec{U}\right)\vec{\nabla }f+\frac{R}{3}\left(\vec{\nabla }\cdot \vec{U}\right)\frac{\partial f}{\partial R},$$

where $f=f(\vec{r},R,t)$ is an omnidirectional distribution function of three spatial coordinates $\vec{r}=r\left(r,\theta ,\varphi \right)$, *r*—radial distance, θ—heliolatitude, φ—heliolongitude, *R*—magnetic rigidity and *t*—time. $\vec{U}$ is the solar wind velocity, *K* is the anisotropic diffusion tensor of the GCR particles, $K^{T}$ its transpose.

Based on the Ito formulation the set of the stochastic differential equations (SDEs), corresponding to the Equation (1), can be solved (e.g., [18]). In the case of the GCR transport the set of SDEs in three dimensional heliocentric spherical coordinate system have a form:

(2) $$\begin{array}{ccc} dr & = & \left(\frac{2}{r}K_{rr}^{S}+\frac{\partial K_{rr}^{S}}{\partial r}+\frac{ctg\theta }{r}K_{\theta r}^{S}+\frac{1}{r}\frac{\partial K_{\theta r}^{S}}{\partial \theta }+\frac{1}{rsin\theta }\frac{\partial K_{\varphi r}^{S}}{\partial \varphi }+U+v_{d,r}\right)\cdot dt+{\left[B\cdot dW\right]}_{r}\\ d\theta & = & \left(\frac{K_{r\theta }^{S}}{r^{2}}+\frac{1}{r}\frac{\partial K_{r\theta }^{S}}{\partial r}+\frac{1}{r^{2}}\frac{\partial K_{\theta \theta }^{S}}{\partial \theta }+\frac{ctg\theta }{r^{2}}K_{\theta \theta }^{S}+\frac{1}{r^{2}sin\theta }\frac{\partial K_{\varphi \theta }^{S}}{\partial \varphi }+\frac{1}{r}v_{d,\theta }\right)\cdot dt+{\left[B\cdot dW\right]}_{\theta }\\ d\varphi & = & \left(\frac{K_{r\varphi }^{S}}{r^{2}sin\theta }+\frac{1}{rsin\theta }\frac{\partial K_{r\varphi }^{S}}{\partial r}+\frac{1}{r^{2}sin\theta }\frac{\partial K_{\theta \varphi }^{S}}{\partial \theta }+\frac{1}{r^{2}sin^{2}\theta }\frac{\partial K_{\varphi \varphi }^{S}}{\partial \varphi }+\frac{1}{rsin\theta }v_{d,\varphi }\right)\cdot dt+{\left[B\cdot dW\right]}_{\varphi }\\ dR & = & -\frac{R}{3}\left(\vec{\nabla }\cdot U\right)\cdot dt, \end{array}$$

where $\vec{r}$ is the trajectory of individual pseudoparticle in the phase space and $dW_{i}$ is the Wiener process. The $v_{d,i}=\frac{\partial K^{A}}{\partial x_{j}}$ denotes the drift velocity, where $K^{A}$ is the antisymmetric part of the full 3D anisotropic diffusion tensor of GCR particles containing the symmetric $K^{S}$ and antisymmetric $K^{A}$ part $K=K^{S}+K^{A}$, presented in detail in [19]. The matrix $B_{ij}$, (i,j=r,θ,φ) has a form [16,20]:

$$B_{ij}=\left[\begin{array}{ccc} \sqrt{2K_{rr}^{S}} & 0 & 0\\ \frac{\frac{2K_{r\theta }^{S}}{r}}{\sqrt{2K_{rr}^{S}}} & \sqrt{2\frac{K_{\theta \theta }^{S}}{r^{2}}-\frac{{\left(\frac{2K_{r\theta }^{S}}{r}\right)}^{2}}{2K_{rr}^{S}}} & 0\\ \frac{\frac{2K_{r\varphi }^{S}}{rsin\theta }}{\sqrt{2K_{rr}^{S}}} & \frac{\frac{2K_{\theta \varphi }^{S}}{r^{2}sin\theta }-\frac{\frac{2K_{r\theta }^{S}}{r}\frac{2K_{r\varphi }^{S}}{rsin\theta }}{2K_{rr}^{S}}}{B_{\theta \theta }} & \sqrt{2\frac{K_{\varphi \varphi }^{S}}{r^{2}sin^{2}\theta }-B_{\varphi r}^{2}-B_{\varphi \theta }^{2}} \end{array}\right].$$

Solving the Equation (1) by the time-backward approach, we initialize the pseudoparticles from the point of interest, e.g., Earth orbit by fixing their rigidity, initial position (radial distance, heliolongitude, and heliolatitude) and trace their trajectory backward-in-time until crossing the heliospheric boundary. During their travel throughout the heliosphere, the pseudoparticles gain/lose their energy/rigidity proportionally to the travel time. The set of Equations (2) bounds this trajectory in conjunction with their rigidity. Value of the distribution function is assumed as the statistical mean among the values of a local interstellar function for all simulated pseudoparticles. The more details on the solution of the set SDEs (2) can be found in [16,17,21].

In this paper, we assume that Fd takes place due to established corotating heliolongitudinal disturbances in the interplanetary space encountered by the pseudoparticles during their travel through the heliosphere. We assume that this region is restricted in space (r<30AU and $90^{\circ }\leq \varphi \leq 270^{\circ })$ and travels with solar wind. Inside this region, the increase of IMF turbulence as a result of shock-wave transported from the Sun by the solar wind, causes the reduction of the diffusion coefficient of GCR particles. We simulate this process by the gradual decrease and then the increase of the diffusion coefficient $K_{\left|\right|}$ of cosmic ray particles having a form:

(3) $$K_{\left|\right|}=K_{0}\cdot K\left(r\right)\cdot K\left(R,\gamma \right).$$

The first factor K(r)=1+0.5·(r/1AU) reflects the change of $K_{\left|\right|}$ with distance from the Sun, as is usually applied in the literature (e.g., [8]). The second $K\left(R,\gamma \right)=R^{\gamma }$ describes the exponential changes of the diffusion coefficient versus particles rigidity *R*. We assume that the exponent γ decreases in the vicinity of space as the result of the increased IMF turbulence [8,9,10]. In result, the initial trajectory of pseudoparticles is disturbed by the shock wave causing the fewer pseudoparticles to reach the Earth orbit. Additionally, we assume rigidity dependence of the exponent γ in the following form:

(4) $$\gamma \left(R\right)=\{\begin{array}{c} \alpha -\beta \cdot \tau^{\delta }\cdot exp\left(\eta \cdot \tau \right)\cdot R^{\zeta };\;within\;disturbed\;region,\\ \alpha ;\;\;\;\;\;outside\;disturbed\;region. \end{array}$$

The τ=0 corresponds to the time when the pseudoparticles encounter the disturbed region. We threat the parameters α, β, δ, η and ζ as unknown because so far no measurable data is allowing us to precisely estimate their values. The only physical requirement is 0<γ(R)<2 (e.g., [12]). The parameter α describes the initial level of diffusion, β defines its relative change, parameter δ reflects how fast the exponent γ decreases, while the parameter η defines the recovery time. The rigidity dependence of the exponent γ(R) defines the parameter ζ.

**Figure 1:**
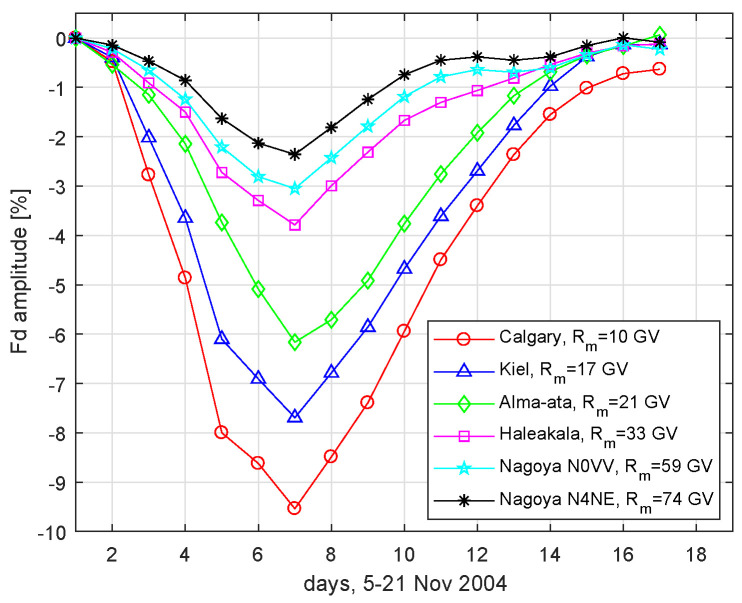
The daily amplitudes of Fd observed in the period of 5–21 November 2004 by the Calgary, Kiel, Alma-Ata, Haleakala neutron monitors and two channels of the Nagoya ground muon telescope.

In the next section, we present how these values are estimated by the ABC method so that the amplitudes of the Fd stochastic model matched the observational amplitudes of Fd registered in November 2004 (see Figure 1) by the neutron monitors and muon telescopes.

### 2.2. Approximate Bayesian Computation Algorithm

The ABC methods, also known as likelihood-free methods have appeared in the past fifteen years as the most satisfactory approach to intractable likelihood problems, first in genetics (e.g., [22]) then in a broader spectrum of applications (for review see [23]). This computational methods allow us to choose the set of parameters of considered scenario (model) not deterministically, but sampled from a probability distribution. The data generated by simulation are then reduced to summary statistics, and the sampled parameters are accepted or rejected on the basis of the distance between the simulated and the observed data.

Let’s assume λ to be a parameter vector, given the prior distribution π(λ). The goal of Bayesian inference is to approximate the posterior distribution, $\pi \left(\lambda |d_{obs}\right)\propto \pi \left(d_{obs}|\lambda \right)\pi \left(\lambda \right)$, where $\pi (d_{obs}|\lambda )$ is the likelihood of λ given the observed data $d_{obs}$. The main idea of ABC methods is to accept λ as an approximate posterior draw if the associated generated data *d* is close enough to the observed data $d_{obs}$. Accepted parameters are a sample from $\pi (\lambda |\rho \left(d,d_{obs}\right)<\epsilon )$ where $\rho (d,d_{obs})$ is the chosen measure of discrepancy between *d* and $d_{obs}$; ϵ is a threshold defining ’closeness margin’. If ϵ is sufficiently small then the distribution $\pi (\lambda |\rho \left(d,d_{obs}\right)<\epsilon )$ will be a good approximation for the posterior distribution $\pi (\lambda |d_{obs})$. It is often difficult to define an adequate distance function $\rho (d,d_{obs})$ between the simulated and observed data, mainly when they lay in the space of high dimension. To overcome this curse of dimensionality distance function is replaced with a distance defined by summary statistics, $\rho (S\left(d\right),S\left(d_{obs}\right))$.

In ABC Sequential Monte Carlo (SMC) method, the set of samples with weights, called particles sampled from the prior distribution $\pi (\lambda^{1})$, is propagated through a sequence of intermediate posterior distributions $\pi (\lambda^{t}|\rho \left(d,d_{obs}\right)<\epsilon_{t})$, t=1,…,T, until it represents a sample from the target distribution, $\pi (\lambda^{T}|\rho \left(d,d_{obs}\right)<\epsilon_{T})$. These methods aim to generate draws from $\pi (\lambda^{t}|\rho \left(d,d_{obs}\right)<\epsilon_{t})$, at each of a series of sequential steps *t*, where $\epsilon_{t}$ defines a series of thresholds.

The Algorithm 1 presents the subsequent steps of the applied ABC SMC algorithm. In the first step the threshold schedule $\epsilon_{1}>\epsilon_{2}>,\ldots ,>\epsilon_{T}$ is initialized. Then *N* samples are simulated based on the predefined a priori distribution $\pi (\lambda^{1})$ and the corresponding acceptance condition $\rho \left(d^{Fd_{data}},d_{obs}^{Fd_{data}}\right)<\epsilon_{1}$. Next, the initial uniform weights are calculated. Samples, denoted by a tilde are drawn from the previous generation with probabilities $w_{j}^{t-1}$. Using perturbation kernel $K_{\lambda ,t}\left(\lambda_{i}^{t}|{\tilde{\lambda }}_{i}\right)$ new ‘fresh’ samples $\lambda_{i}^{t}$ are obtained, with the veracity of the condition $\rho \left(d^{Fd_{data}},d_{obs}^{Fd_{data}}\right)<\epsilon_{t}$. The weights are calculated according to the formula in stage (22). In stage (23) the weights are normalized and the time-step is increased: t=t+1. This procedure is repeated until t≤T.

In this paper the scanned parameters vector λ is

(5) λ≡(α,β,δ,η,ζ).

These parameters define the profile of the diffusion coefficient given by Equation (3). The initial knowledge about the probable profile of $K_{\left|\right|}$ allowed us to declare the following priori distribution on particular parameters:

(6) $$\pi \left(\lambda^{1}\right)\equiv \left\{\begin{array}{c} \alpha ∼U(0.2,2)\\ \beta ∼U(0,0.1)\\ \delta ∼U(0.5,2.5)\\ \eta ∼U(-0.05,0.05)\\ \zeta ∼U(-1,0) \end{array}\right.$$

To estimate the fit of the Fd model to the observations as a distance measure we used normalized approximation error between all the data. We have slightly modified the classical Fractional Bias (FB) measure $\rho (d,d_{obs})$ as:

(7) $$\rho \left(d^{Fd_{data}},d_{obs}^{Fd_{data}}\right)=\frac{1}{K}\sum_{k}^{K}\left(\frac{1}{NM}\sum_{R}^{NM_{s}}\frac{|I_{R}^{k}-{\hat{I}}_{R}^{k}|}{I_{R}^{k}+{\hat{I}}_{R}^{k}}\right),$$

where *K* is a number of used Fd data samples (days), NM is a number of used neutron monitor stations corresponding in the model to various rigidities, ${\hat{I}}_{R}^{k}$—is the Fd amplitude reported by *R*-th neutron monitor on *k*-time (day) and $I_{R}^{k}$—corresponding modelled Fd amplitude. The measure $\rho (d^{Fd_{data}},d_{obs}^{Fd_{data}})$ estimates the averaged, both in the pseudoparticles rigidity and time, normalized relative discrepancy between the modeled and registered amplitudes of Fd.

**Algorithm 1** ABC SMC
1:Initialize threshold schedule each $\epsilon_{1}>\epsilon_{2}>,\ldots ,>\epsilon_{5}$2:Initialize Forbush threshold schedule $Fd_{data}$, Fd={1,2,4,7,15};3:Set t=14:**for**i=1 to *N* **do**5:  Setup data set $Fd_{data}=\left\{1,L\right\}$, L=17-Fd data duration6:  Draw additional Fd[t]-numbers from set {2,3,…,L−1} to the $Fd_{data}$ set7:  Simulate $\lambda_{i}^{t}∼\pi \left(\lambda^{t}\right)$8:  $d^{Fd_{data}}∼\pi \left(\cdot |\lambda_{i}^{t}\right)$9:  Until $\rho \left(d^{Fd_{data}},d_{obs}^{Fd_{data}}\right)<\epsilon_{t}$10:  Set $w_{i}^{t}=\frac{1}{N}$11:
**end for**
12:**for**t=2 to *T* **do**13:  **for** i=1 to *N* **do**14:   Pick ${\tilde{\lambda }}_{i}$ from the set ${\left\{\lambda_{j}^{t-1}\right\}}_{1\leq j\leq N}$15:      with probabilities ${\left\{w_{j}^{t-1}\right\}}_{1\leq j\leq N}$16:   Draw $\lambda_{i}^{t}∼K_{\lambda ,t}\left(\lambda_{i}^{t}|{\tilde{\lambda }}_{i}\right)$17:   Setup set $Fd_{data}=\left\{1,L\right\}$18:   Draw additional Fd[t]-numbers from set {2,3,…,L−1} to the $Fd_{data}$ set19:   $d^{Fd_{data}}∼\pi \left(\cdot |\lambda_{i}^{t}\right)$20:   Until $\rho \left(d^{Fd_{data}},d_{obs}^{Fd_{data}}\right)<\epsilon_{t}$21:   Compute new weights as22:      $w_{i}^{t}\propto \frac{\pi (\lambda_{i}^{t})}{\sum_{j}w_{j}^{t-1}K_{\lambda ,t}\left(\lambda_{i}^{t}|\lambda_{j}^{\left(t-1\right)}\right)}$23:   Normalize weights $w_{i}^{t}$ for i=1,…,N24:  **end for**25:
**end for**



#### 2.2.1. Threshold Schedule Updating Scheme

The most commonly used adaptive scheme for threshold choice is based on the quantile of the empirical distribution of the distances between the simulated data and observations (e.g., [24,25]). This method determines $\epsilon_{t}$ in the *t* time-iteration by sorting the measure ${\left\{\rho_{\lambda^{i}}^{t-1}\left(d^{Fd_{data}},d_{obs}^{Fd_{data}}\right)\right\}}_{1<i\leq N}$ and setting $\epsilon_{t}$ such that they fulfil the predetermined percent $Perc_{t}$. This method defines the $Perc_{t}$ percentile as the value below which $Perc_{t}\cdot 100\%$ of the probability mass can be found. The value of the quantile is judged based on the previous step t−1, but with two major improvements:

- The distribution of ${\left\{\rho_{\lambda^{i}}^{t-1}\right\}}_{i=1,\ldots ,N}$ measure of samples set is discrete, thus some approximation is required. To keep the procedure flexible and support the interval [0,1] the best choice for continuous distributions seems to be the family of Beta distributions.
- We propose to define the value of $Perc_{t}$ to be dependent on the ratio between a number of currently accepted samples (AS) and the number of all hitherto generated samples (AGS) i.e., $Perc_{t}=1-AS/AGS$.

#### 2.2.2. Transition Kernel

The methods for construction of the perturbation kernel required in the ABC SMC approach are discussed in [26]. The computational efficiency of different kernels, in terms of acceptance rates and computational cost, is estimated based on the number of the required simulations. Many proposals were examined, e.g., uniform, normal component-wise, *M* nearest neighbors, OLCM (Optimal Local Covariance Matrix), FIM (Fisher Information Matrix) scaled based on the whole population, FIM scaled based on nearest neighbors. Authors showed that for many complicated posterior distributions, locally adapted kernels tend to show the best performance. The most efficient version of kernel in term of number of simulations and running time occurred the one using the *M* nearest neighbours. Thus, in presented in this paper algorithm we have chose the multivariate normal kernel with *M* nearest neighbors. The multivariate normal kernel with *M* neighbours follows the rule: for each particle $\lambda \in \{\lambda_{i}^{t-1},1\leq i\leq N\}$, the *M*-nearest neighbours of λ are selected, and the perturbed particle is sampled according to a multivariate normal distribution of mean equal λ and the empirical covariance $\Sigma_{\lambda ,M}^{t}$ based on the *M* nearest neighbours of λ. The cost of local measures based on *M* nearest neighbors may seem high to consider their use in typical toy examples. However, given the increase in acceptance rate that authors have observed, and the generally high computational cost of the forward model, they can prove to be fruitful and extremely beneficial for our very costly computational model. The authors in [26] pay attention to the disadvantages of choosing this perturbation kernel. Its efficiency strongly depends on the chosen value of *M*. Too small value of *M* may lead to a lack of exploration of parameter space, while too large would offer little or no advantage compared to the standard multivariate normal kernel. In our case, the number of samples allocated to one time-step is N=1000 samples. Based on pre-processing experiments we have selected M=100 and Euclidian distance in our nearest neighbors procedure.

## 3. Results

We employed the ABC SMC methodology to estimate the vector parameters λ (Equation (6)) of the diffusion coefficient $K_{\left|\right|}$ (Equation (3)) by fitting the Fd model output to the Fd observed on 5–21 November 2004. The more details on this event can be found in [10]. As the reference data, we have selected the data from four neutron monitors and two channels of Nagoya muon telescope. The daily amplitudes of the Fd registered by these detectors are presented in Figure 1. In the stochastic model of the Fd as the pseudoparticles initial rigidity *R* we have assumed the value equal to the median rigidity of response $R_{m}$ of the detector to which we will compare the model output by the Formula (6). We have adopted stations $R_{m}$ values as was given in [27] i.e., Calgary $R_{m}=10$ GV, Kiel $R_{m}=17$ GV, Alma-Ata $R_{m}=21$ GV, Haleakala, $R_{m}=33$ GV, Nagoya N0VV $R_{m}=59$ GV and Nagoya N4NE $R_{m}=74$ GV.

**Figure 2:**
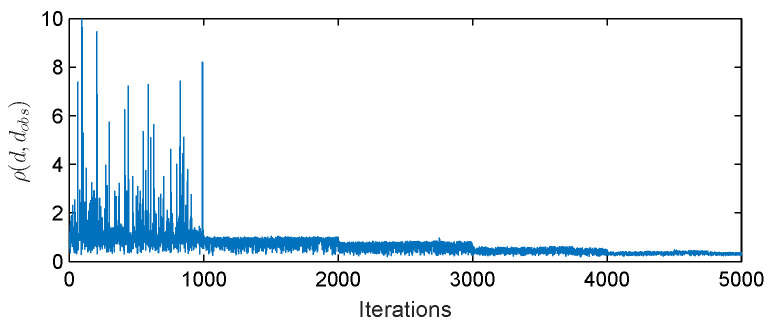
The changes of the distance measure $\rho (d^{Fd_{data}},d_{obs}^{Fd_{data}})$ for all accepted samples in subsequent ABC SMC iterations.

**Figure 3:**
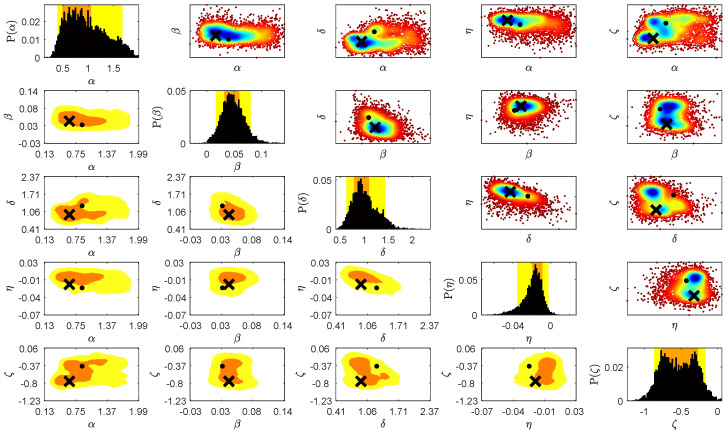
The bivariate and marginal posterior distributions for all searched parameters λ≡(α,β,δ,η,ζ). Probability density colors the plot, the more blue regions, the higher is its probability. The lower diagonal plots presents the 50% and 90% credible regions marked by orange and yellow color, respectively. The cross marks the value of parameter with the higher probability. The black dot marks the set of parameters with the lowest distance measure $\rho (d^{Fd_{data}},d_{obs}^{Fd_{data}})$.

**Figure 4:**
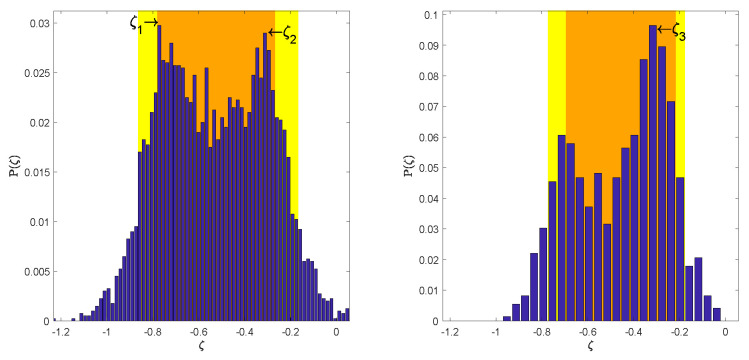
The marginal posterior distributions for ζ parameter. The right panel presents the distribution estimated based on the filtered samples with distance measure $\rho (d^{Fd_{data}},d_{obs}^{Fd_{data}})<0.33$.

**Figure 5:**
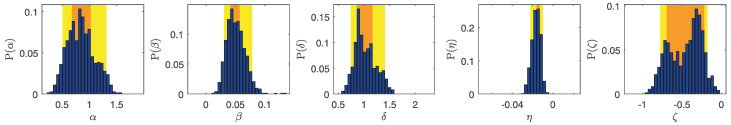
The marginal posterior distributions for all searched parameters λ≡(α,β,δ,η,ζ) based on the filtered samples $\rho (d^{Fd_{data}},d_{obs}^{Fd_{data}})<0.33$.

The space of parameters λ (Equation (5)) was sampled by the Algorithm 1. Each sample required the run of the Fd model described in Section 2.1 for six pseudoparticles rigidities corresponding to stations $R_{m}$ values. The amplitude of Fd on each day was calculated with respect to the intensity on the first day (5 November 2004). Figure 2 presents the changes of the distance measure calculated by the Formula (6) for each accepted sample λ. This value reflects how well the modeled amplitudes of Fd for all six rigidities agree with the recorded one. In the first time-step of Algorithm 1 the distance measure was estimated comparing the amplitudes during three days, in the second time-step five days, while at the final fifth time-step the distance measure covers all days of the Fd. Apart the first and last day of the Fd in the intermediate time-steps, the days, in which the comparison was made, were drawn randomly. The distance $\rho (d^{Fd_{data}},d_{obs}^{Fd_{data}})$ is decreasing in subsequent time-steps of the ABC SMC algorithm reaching in the last stage the mean value ∼0.33. The bivariate and marginal posterior distributions for all searched parameters λ≡(α,β,δ,η,ζ) are presented in Figure 3. On the main diagonal are presented the marginal posterior distributions for each parameter. The upper diagonal plots visualize the bivariate distributions. The more blue region the higher is a probability. The lower diagonal plots visualize the 50% and 90% credible region. The values of the parameter with the highest probability are denoted by the cross, while the dot represents the set of parameters λ for which the minimal value od distance measure (6) was reached. All summary statistics are given in the Table 1. As the estimated parameter value we provide the central value of the histogram bar with highest probability and as the error the half of the bar width. The most unambiguous distributions were attained for the parameters β, δ and η. The distributions characterize high kurtosis and relatively narrow confidence intervals. Analysis of parameter α distributions suggests more than one highly probable value. This is understandable because the parameter α determines the initial level of diffusion at the beginning of the Fd and is not crucial for Fd amplitude. The most interesting results were achieved for the parameter ζ, which determines the rigidity dependence of the exponent γ. Its distribution is bimodal. The more detailed analysis of ζ posterior distribution is presented in Figure 4. Left panel in Figure 4 shows that the ABC SMC algorithm pointed two values of the ζ as equally probable i.e., $\zeta_{1}=-0.7623\pm 0.0085$ and $\zeta_{2}=-0.3027\pm 0.0085$. To analyze this distribution in more detail, we decided to exclude from the distribution the samples with high distance measure values. As far as, in the final time step the distance measure takes into account all days of the Fd, and the parameters estimation should be the best, as the filter upper limit value we have assumed the mean distance value in the final time-step, i.e., 0.33. This method allowed to extract the set of samples for which the distance measure is smaller than 0.33. Based on this set we have estimated the marginal posterior distribution again. The results are presented in the right panel of Figure 4. Now the right mode of the distribution become decisive, and the most probable is $\zeta_{3}=-0.2970\pm 0.0199$, which agrees in the scope of error with $\zeta_{2}$. Results of the same filtering procedure performed for all other parameters are presented in Figure 5. We can see that filtering allowed us to exclude multimodality also for the parameter α, while the results for other parameters agree in the scope of error with the previous one. The corresponding values are given in Table 1.

**Figure 6:**
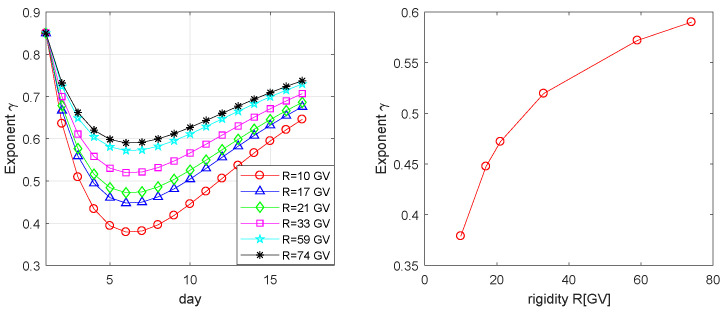
The profile of the exponent γ calculated by the Formula (4) with use of the estimated by the ABC SMC algorithm values of parameters $\lambda^{MAP_{f}}$ (Table 1).

Summarizing, we can conclude that the most probable values of searched parameters are $\lambda^{MAP_{f}}\equiv \left\{\alpha =0.8501\pm 0.0288,\beta =0.0465\pm 0.0027,\delta =0.9206\pm 0.0303,\eta =-0.0130\pm 0.0015,\zeta =-0.2970\pm 0.0199\right\}$. We want to underline that the value of parameter ζ is not zero. It means that the data confirm that during the Fd indeed there exists the dependence of the Fd rigidity spectrum exponent γ on particles rigidity. The corresponding profile of the exponent γ of the diffusion coefficient $K_{\left|\right|}$ for various pseudoparticles rigidities is presented in Figure 6. Comparison of the temporal profile of the exponent γ and GCR intensities (Figure 1) shows that we observe some shifting in the decreasing phase, i.e., the minimum of γ is observed one day before the minimum of GCR intensity. This theoretical result is in agreement with the temporal profiles of the rigidity spectrum exponent of Fd in November 2004 presented in [10] (Figure 1 in [10]). The right panel of Figure 6 shows the proven by the study rigidity dependence of the exponent γ.

## 4. Summary

The novelty of this work was to employ the ABC SMC method to estimate the most probable parameters of the diffusion coefficient during the Fd of GCR intensity. The formula proposed for the diffusion coefficient $K_{\left|\right|}$ contains the rigidity dependence of the exponent γ being the exponent of the rigidity spectrum of Fd. This dependence was implied by the previous experimental investigations [9,10]. However, the experimental study have some methodical limitations, which impede to derive the specific formulas. Firstly, to estimate the rigidity spectrum exponent γ we require at least data from two detectors with distinguishable rigidity of responses Rm (see e.g., [8]). Secondly, estimation of the daily changes of the exponent ν of the PSD of the IMF turbulence being in relation with γ (γ∝2−ν [8,9,10]) is doubtful. To attain the single value of the exponent ν in low frequency range ($f\in [10^{-6},10^{-5}]$) responsible for the scattering of the GCR particles to which neutron monitor and ground meson telescopes respond methodology requires relatively long data series (at least 12 days, see e.g., [10]), which covers the Fd duration. Thus the only way is to employ the theoretical modeling and try to estimate the required formulas based on a comparison of the model output with the experimental data.

In this paper, we have scanned the diffusion coefficient parameters space by ABC SMC algorithm to estimate the value of parameters giving the best fit of the Fd model with the experimental data. In calculations, we have used the GCR daily intensities recorded by the four neutron monitors and Nagoya muon telescope during the Fd event on 5–21 November 2004. To model the Fd, we have employed the stochastic approach based on the solution of the SDEs corresponding to the Parker transport equation. The performed computations allowed to estimate with reasonable statistics all five parameters of the diffusion coefficient. Moreover, the results presented in this paper confirm that during the Fd in November 2004 there existed the power-law dependence of exponent γ on rigidity in the form $\gamma \left(R\right)\propto R^{-0.3}$. Important is that employed ABC SMC methodology also provided the information that non-existence of the rigidity dependence of exponent γ (i.e., ζ=0) has a zero-probability. The obtained time-profile of the estimated exponent γ agrees with the temporal changes of the rigidity spectrum exponent of the Fd calculated based on the experimental data and presented in [10]. This agreement increases our belief in the correctness of the proposed framework and the achieved results. However, the obtained results are limited to the one specific Fd. We must remember that Fds are quite complicated features and their characteristics may vary depending on the specific situation in the heliosphere during the Fd creation. The future work will be focused on checking whether the estimated rigidity dependence of exponent γ is visible in other registered Fds. If it is a case, the question arises does the rigidity dependence is the same for all Fds, or are there required some conditions to be fulfilled like e.g., the relevant amplitude of Fd, the size of modulation region or the Sun polarity cycle.

## Figures and Tables

**Table 1:** Summary of the essential statistics obtained from the ABC SMC algorithm results. The $\lambda^{MAP}$ denotes the maximum a posteriori estimation, $\lambda^{MAP_{f}}$ denotes the maximum a posteriori estimation from the filtered samples, $\lambda^{⋆}$ parameter set with minimal distance value; $\bar{\lambda }$ mean; σ(λ) standard deviation; var(λ) variance; s(λ) skewness; k(λ) kurtosis; credible interval 90% $CI^{90\%}\left(\lambda \right)$ ; credible interval 50% $CI^{50\%}\left(\lambda \right)$ .

Parameters	α	β	δ	η	ζ
$\lambda^{MAP}$	0.6365 ± 0.0123	0.0411 ± 0.0011	0.9379 ± 0.013	−0.0156 ± 0.0007	−0.7623 ± 0.0085
$P(\lambda^{MAP})$	0.0278	0.0478	0.051	0.0712	0.0297
$\lambda^{MAP_{f}}$	0.8501 ± 0.0288	0.0465 ± 0.0027	0.9206 ± 0.0303	−0.0130 ± 0.0015	−0.2970 ± 0.0199
$P(\lambda^{MAP_{f}})$	0.1033	0.1419	0.1653	0.2562	0.0964
$\lambda^{☆}$	0.8871	0.0292	1.265	−0.0222	−0.3936
$\bar{\lambda }$	0.9752	0.0479	1.0127	−0.0192	−0.5307
σ(λ)	0.39	0.0207	0.2576	0.0104	0.2289
var(λ)	0.1521	0.0004	0.0664	0.0001	0.0524
s(λ)	0.4537	0.4338	0.6272	−0.9545	0.0224
k(λ)	2.3167	3.6615	3.2615	4.3960	2.1279
$CI^{90\%}\left(\lambda \right)$	[0.3901, 1.6716]	[0.016, 0.08]	[0.6261, 1.4316]	[−0.0354, −0.0024]	[−0.8644, −0.1665]
$CI^{50\%}\left(\lambda \right)$	[0.4886, 1.0555]	[0.032, 0.0594]	[0.782, 1.0938]	[−0.0196, −0.009]	[−0.7793, −0.2687]

## Abbreviations

The following abbreviations are used in this manuscript:

ABC	Approximate Bayesian Computation
ABC SMC	Approximate Bayesian Computation with Sequential Monte Carlo
AGS	All hitherto Generated Samples
AS	All Samples
FB	Fractional Bias
Fd	Forbush decrease
GCR	Galactic Cosmic Rays
IMF	Interplanetary Magnetic Field
PSD	Power Spectral Density
SMC	Sequential Monte Carlo
SDE	Stochastic Differential Equation
